# 全肺切除术后并发症及预后风险评估

**DOI:** 10.3779/j.issn.1009-3419.2020.101.06

**Published:** 2020-07-20

**Authors:** 晓康 郭, 化锋 王, 煜程 魏

**Affiliations:** 266000 青岛，青岛大学附属医院胸外科 Department of Thoracic Surgery, The Affiliated Hospital of Qingdao University, Qingdao 266000, China

**Keywords:** 全肺切除术, 并发症, 危险因素, Pneumonectomy, Complications, Risk factors

## Abstract

外科手术是目前根治非小细胞肺癌（non-small cell lung cancer, NSCLC）的最有效方式。全肺切除作为一种术式被应用于临床中。对于中央型肺癌，袖式肺叶切除术因其术后肺功能丧失少、术后并发症及死亡率低逐渐取代全肺切除术成为主流。然而为保证肿瘤学效果，当其他术式无法完全切除时，全肺切除术式仍是必要的。全肺切除术后主要发生心肺并发症，充分了解全肺切除术后相关并发症能帮助临床医师及时做出诊断，并进一步采取相关措施降低术后并发症对患者的不良影响。充分了解预后相关危险因素可帮助临床医师提前采取措施尽可能规避风险，从而改善患者预后。

## Abstract (en)

Surgery is the most effective way to cure non-small cell lung cancer currently. Although sleeve lobectomy, with less reduction of respiratory function and a lower mortality rate, would not compromise oncological results in well-selected patients with central lung cancer, pneumonectomy is still necessary when complete resection could not be achieved by other surgical procedures. Cardiopulmonary complications are the most common complication after pneumonectomy. Fully understanding the related complications after pneumonectomy can help surgeons make a diagnosis timely, and further take relevant measures to reduce the adverse effects of postoperative complications on patients. What's more, in order to avoid postoperative complications and improve the prognosis of patients as much as possible, it is necessary to fully understand the risk factors to minimize the risk and to make the patients benefit from pneumonectomy as much as possible.

目前肺癌为世界发病率和死亡率最高的肿瘤^[1]^。自1933年Graham^[2]^报道第1例行全肺切除术的肺癌病例至今，以手术为中心的综合治疗已然成为非小细胞肺癌（non-small cell lung cancer, NSCLC）的治疗原则，其中外科手术方式多种多样，肺切除术式的历史从全肺切除开始，随着诊断、治疗技术的不断提高，麻醉方式的不断改进，肺叶切除术逐渐取代全肺切除术成为治疗肺癌的标准术式。随着袖式肺叶切除术的逐渐成熟，因其较全肺切除术后并发症少、术后肺功能丧失少和生活质量更好^[3, 4]^，全肺切除术的比例进一步下降。全肺切除术仅占20%左右比例。然而，对于临床上中央型肺癌或较大肿瘤，肺叶切除术、袖式肺叶切除术、支气管肺动脉双袖式肺叶切除术等术式往往无法做到R0切除，而全肺切除术可以提高手术切除率，减少不必要的开胸探查。因此，全肺切除术在肺癌外科治疗中仍具有一定的作用。

全肺切除术作为一种高风险手术方式，相比其他手术方式，往往有更差的短期及长期预后^[4-6]^。充分了解全肺切除术后相关并发症和影响预后的相关危险因素可帮助临床医师积极采取相关措施规避并发症和风险发生，从而改善患者预后。

## 1 全肺切除术后相关并发症

围术期至少发生1种并发症的发生率为21.4%-39.0%，30 d死亡率为3.2%-6.8%，5年生存率为27%-45%（表 1）。为改善患者预后，临床医师充分了解全肺切除术后相关并发症是必要的。全肺切除术后并发症主要包括心血管系统和呼吸系统并发症。

**1:** 全肺切除术治疗NSCLC的围术期结果及5年生存率 Perioperative morbidity and 5-year survival rate of pneumonectomy in NSCLC

Author, year	Case	Overall morbidity (%)	30-day mortality (%)	Morbidity of complications (%)				5-year survival rate (%)
				Pneumonia	ARDS	BPF	Arrhythmia	
Harpole DH *et al*^[7]^, 1996	136	42.0	3.0	5.9	2.9	2.9	24.0a	-
Ludwig C *et al*^[8]^, 2005	194	23.0	4.6	1.5	3.1	3.6	3.6b	27.0
Powell ES *et al*^[9]^, 2009	312	31.7	5.4	-	-	-	19.9	-
Shapiro M *et al* ^[10]^, 2010	1, 267	30.4	5.6	4.7	3.1	0.8	21.2	-
Pricopia C *et al* ^[11]^, 2014	2, 064	21.4	6.2	3.3c	-	2.9	2.9	-
Qadri SS *et al* ^[12]^, 2016	306	-	4.5	-	-	-	-	32.0
Gu C *et al*^[13]^, 2017	406	36.7	3.2	5.9	2.5	2.0	25.4	32.5
Skrzypczak PJ *et al*^[14]^, 2019	1, 160	56.7	4.0	-	-	8.9	31.8	45.0
ARDS: acute respiratory distress syndrome; BPF: bronchopleural fistula；“-”: no data；^a^: supraventricular arrhythmia; ^b^: cardiac complications; ^c^: pneumonia and ARDS; NSCLC: non-small cell lung cancer.								

### 1.1 心血管系统并发症

#### 1.1.1 心律失常

全肺切除术后最常见的心律失常为室上性心律失常，其中心房颤动（房颤）最为常见。房颤发生率约为4%-25%^[5, 7-16]^。术后房颤的发生机制是复杂的，目前尚未完全明确。非心脏胸腔手术术后房颤由基础心脏情况、手术情况、术后情况三方面共同决定^[15]^。手术操作过程中对胸腔内植物神经影响、心房扩张或受到急性牵拉、心包炎症反应、电解质紊乱、氧化应激等均可诱发房颤，术后交感神经兴奋性增强、炎症也是房颤的危险因素^[16]^。有研究^[17]^结果表明，胸腔镜下全肺切除的患者术后房颤发生概率高于开放全肺切除患者，且存在统计学差异。他们认为由于腔镜组心包内处理中央血管的比例明显高于开放组所致，心包内操作是术后房颤的危险因素。Harpole等^[7]^研究结果表明，高龄、心包内操作、胸膜外全肺切除术、右全肺切除术、术后并发症均为房颤的危险因素。术前服用β受体阻断剂及胺碘酮对术后房颤有着良好的预防作用，但可能会带来药物相关不良反应。当患者出现术后房颤时，首先控制心率还是心律以及是否需要抗凝仍存在一定争议^[18-21]^。

#### 1.1.2 肺动脉高压及右心功能不全

全肺切除术后发生肺动脉高压及右心功能不全的机制为健侧肺血流量的增加引起肺动脉高压，严重的肺动脉高压可引起右心室后负荷增加，当心脏失代偿时导致右心功能不全^[22]^。在临床工作中，全肺切除术后右心功能不全的发生率较低。我们之前的研究表明，术后右心功能不全的发生率仅为3.4%。Shapiro等^[23]^研究结果表明，1, 264例全肺术后患者中也仅有24例（1.9%）患者发生心力衰竭。除肺血流量增加外，低氧血症、酸中毒、高碳酸血症、交感神经兴奋等诱因也可以引起肺动脉压力升高^[24]^。Deslauriers等^[25]^分析了523例全肺切除患者，术后肺动脉高压发生率虽然高达38%，但仅仅为轻至中度肺动脉高压。Cryer等^[26]^通过动物实验表明：全肺切除合并失血性休克两者联合作用引起肺动脉压力增加较单一作用显著，且右心功能不全的发生率较高。我们认为大多数全肺切除术后仅有流经肺血流量增加这一单一危险因素引起肺动脉压力升高，其余危险因素发生率低，从而降低了右心功能不全的发生风险。

#### 1.1.3 纵隔移位

全肺切除术后胸膜腔内压力的变化是纵隔移位的直接影响因素。术后充分地引流胸膜腔内液体及气体时，会导致胸膜腔内压力变小，纵隔向患侧移位，对侧肺过度膨胀，肺功能受到影响^[27-37]^，还可引起肺水肿、心律失常、心脏疝等并发症，从而增加了围术期死亡风险^[38]^。当术后胸膜腔内液体过多时（胸腔内出血、淋巴液渗出）可导致纵隔偏向健侧，压迫肺组织，影响肺功能。当纵隔过度移位时，可能会导致呼吸功能衰竭，当发生腔静脉扭曲时会导致循环衰竭甚至死亡^[39]^。目前，全肺切除术后胸膜腔的管理有放置引流管和不放置引流管两种理念。这两种方式各有利弊^[39, 40]^。当术后不放置引流管时，经常需要通过胸腔穿刺抽吸气体保持纵隔居中。抽吸完气体后立即行床边胸片，评估纵隔位置，必要时可反复抽吸^[41]^。当术后胸腔内放置引流管时，可通过长期夹闭定期开放来调节胸膜腔内压，保持纵隔居中。一般术后24 h内，术侧膈肌开始逐渐抬高，肋间隙逐渐收缩，纵隔开始偏向术侧。若引流管持续开放，气体从胸腔内连续排出，纵隔向术侧偏移，严重者可引起不良后果^[40]^。目前一些研究^[42, 43]^表明，全肺切除术后即使不夹闭引流管，术后发生纵隔移位等并发症的风险与夹闭无异。

#### 1.1.4 心脏疝

医源性、创伤性、先天性心包缺损是引起心脏疝的主要危险因素^[27]^。心包内全肺切除术可造成医源性心包缺损，进而可引起心脏疝。剧烈咳嗽、剧烈活动、纵隔摆动、体位改变常为心脏疝的诱发因素。自1948年报道首例心脏疝以来^[28]^，全球仅仅报道了60例左右，且心脏疝发生时的临床表现及严重程度不一，目前认为与心包缺损的大小相关^[29-31]^。当心包部分损失时，在上述诱发因素影响下可能会发生心脏疝。较轻的症状常常表现为胸痛、呼吸困难、晕厥等，严重时可发生明显血流动力学变化，甚至引起死亡。Zhao等^[32]^报道了1例行心包内全肺切除患者因手术结束时搬动患者改变体位突然发生严重低血压、心脏骤停。急症开胸探查可见心尖与胸壁左侧毗邻，疝出心脏将上腔静脉和残余心包扭曲到动脉圆锥的左侧。虽然心脏疝在临床工作中发生率低，但存在潜在致死风险。为防止术后心脏疝发生，手术者往往采用扩大心包缺损或心包修补来处理部分心包缺损。扩大心包缺损并不能完全避免心脏疝发生，尤其当破损边缘钙化时，仍有心脏疝发生风险^[30]^。心包修补在防止发生心脏疝的同时，还避免了心包液的丢失。许多研究^[33-35]^证明心包液在维持心脏正常生理活动过程中起着重要作用。对于术中未行处理的部分心包缺损患者，术后预防心脏疝发生的关键是避免剧烈运动、身体姿势突然变化和剧烈咳嗽等诱因发生。对于术后伴严重症状的心脏疝往往需要立即再次进行积极外科干预。但外科干预并非唯一处理方式。2018年，Hu等^[36]^报道了1例右全肺术后心脏疝的患者，患者出现严重的低血压，作者通过向患者患侧胸腔内注入2, 000 mL生理盐水，低血压症状立即改善，患者心脏恢复至正常位置。

#### 1.1.5 心肌梗死

心肌梗死术后发生率约0.2%-2.1%^[37-46]^，术前冠状动脉粥样硬化性疾病为心肌梗死的危险因素^[47]^。

### 1.2 呼吸系统并发症

由于全肺切除术后，肺功能储备明显受限，当发生呼吸系统并发症尤其是急性呼吸窘迫综合征时，围术期死亡风险较高。

#### 1.2.1 肺炎

肺炎是呼吸系统最常见的并发症。既往研究^[7, 8, 10, 11, 13]^表明全肺切除术后肺炎发生率在2%-10%之间。肺炎通常是由于术后疼痛控制不力、行动不便或机械通气时间过长所致。全肺切除术后肺炎的患者生存预后较无肺炎患者差。Simonsen等^[48]^收集了7, 479例包括全肺切除在内的行肺切除手术的肺癌患者，结果表明术后患肺炎患者的30 d内死亡率及30 d-5年死亡率均高于术后未患肺炎患者。为了降低患肺炎的风险，我们的处理措施包括术前戒烟、术后早期和频繁活动、物理疗法促进排痰、开胸术后积极疼痛管理以及必要时纤维支气管镜吸痰等。

#### 1.2.2 脓胸和支气管胸膜瘘（bronchopleural fistula, BPF）

脓胸在全肺切除术后发生并不常见，全肺切除术后脓胸的发生率在2%-12%^[49-52]^。脓胸患者往往有着较高的围术期死亡率^[49]^。高达80%的脓胸患者伴有BPF^[50]^，脓胸伴BPF患者死亡率高达30%-40%，而不伴BPF的脓胸患者死亡率可下降5%^[51]^。脓胸可发生于术后数天至数年内，早期脓胸患者主要表现为持续低热和白细胞计数增高^[52]^。Icard等^[53]^研究结果表明，血清C反应蛋白持续升高或继发水平超过100 mg/L对全肺切除术后脓胸诊断具有非常高的灵敏度（100.0%）和特异度（91.4%）。胸部计算机断层扫描（computed tomography, CT）等影像学检查提示胸腔积液中含有气泡、液气平征象也常常作为怀疑脓胸发生的依据。当怀疑脓胸时，可进一步应用纤维支气管镜探查支气管残端是否存在小瘘口。当术后胸腔内持续漏气（> 7 d），特别是轻度漏气，而且术后复查胸片提示同侧胸膜腔中的积液水平较前降低或在支气管残端高度出现新液气平（“半月板征”）时，应增加对小的支气管胸膜瘘的怀疑^[54]^。当瘘口逐渐变大时，胸腔积液可通过瘘口至对侧肺内，引起对侧肺炎，严重者可发生急性呼吸窘迫综合征。处理术后早期脓胸的原则主要包括胸膜腔充分引流、合理应用抗生素、消除残余脓腔和营养支持等。对于单纯脓胸的患者推荐采用大口径（32 F-36 F）引流管，患者尽量保持反特伦德伦伯格体位。脓胸合并BPF患者，脓液吸入对侧肺内是术后死亡的主要原因^[55]^。患者术后应尽量保持患侧胸腔处于较低位置。当需要机械通气时，应考虑对健侧进行选择性插管，以最大程度地减少对支气管残端的气压伤。而且BPF保守治疗自行愈合概率也较低，外科干预封堵瘘口是有效的方式。若支气管残端过长，可直接切至支气管根部，然后再对残端进行缝合，也可直接采用切割闭合器闭合。如果残端已经位于支气管根部，则需要使用可吸收的缝合线将其间断缝合，重新闭合，然后覆盖带蒂瓣或游离瓣^[52, 53, 56, 57]^。瓣一般来源于肋间肌、心包脂肪垫蒂、前锯肌、大网膜、壁层胸膜、奇静脉和纵隔胸膜等。随着内镜技术的发展，纤维支气管镜下封堵瘘口开始应用于临床，Ueno等^[58]^研究结果表明，传统外科治疗与内镜治疗在支气管胸膜瘘治愈率上无统计学差异，功能状态评分差、术前合并症的存在，肺切除距支气管胸膜瘘诊断的间隔时间短以及活动性肺炎的存在对治疗效果会产生负面影响。其他一些研究^[59, 60]^也证明了内镜技术的安全性及有效性。有研究^[61]^总结了数十年来关于纤维支气管镜技术应用于BPF的进展，其认为，内镜技术因其相对较小的创伤而备受青睐，这对于具有高手术风险的患者具有巨大的价值。但目前仍仅局限于回顾性或小的前瞻性研究，且研究结果差异较大，其有效性仍存在争议。所以，外科手术治疗BPF仍为金标准。

#### 1.2.3 全肺切除后综合征（postpneumonectomy syndrome, PPS）

由于未知的原因，极少数接受全肺切除的患者术后纵隔和心脏向患侧胸腔过度移位，大血管发生扭转，气管、主支气管和肺动脉受到后面的椎骨或主动脉压迫，产生中央气道受压和肺动脉高压相关症状^[62]^。儿童和成年人均可发生。目前认为通过手术纠正移位是最有效的方式。手术方式包括将心包固定在胸骨后部、胸腔置入假体、六氟化硫注入胸腔和盐水填充的乳房假体置入胸腔等^[63]^。随着生物技术、组织工程及3D打印新兴技术崛起，Wang等^[64]^采用立体碳纤维植入治疗PPS。因其与周围器官良好的生物相容性取得了良好的临床效果。PPS除引起中央气道和肺动脉受压外，还可引起食管受压。有研究^[65]^报道了1例右全肺切除术后纵隔过度移位导致食管受压的病例。患者主诉为吞咽困难，胸部CT和胃镜检查均清楚地显示食管因纵隔过度移位受到上腔静脉和降主动脉外在压迫，而没有任何腔内病变。有研究^[66, 67]^表明，全肺切除术后食管运动障碍是由于上下括约肌舒张压增加、食管中部和远端食管蠕动异常或胃排空延迟引起。纵隔移位、局部缺血、食管壁直接损伤、迷走神经损伤或自主神经丛障碍是已知的原因。这种因食管受压引起运动障碍的病例是罕见的，之后再无相关报道，而且也没有相关治疗报道。无论中央气道受压还是食管受压，纵隔过度移位是其根本原因，我们可以推测，PPS的治疗方案可能对缓解食管受压也有效。

## 2 影响预后相关危险因素

全肺切除因较其他术式具有更高的围术期并发症发生率及死亡率^[68]^，而且术后生活质量和远期预后也较差。常常作为最后的手术方式选择。为使全肺切除患者尽可能受益，手术医师全面了解和规避影响预后的危险因素是必要的。

既往研究^[11, 12]^常将年龄视为危险因素，年龄越大，围术期死亡率更高，长期生存率更低。然而一些研究^[13, 69]^结果持不同观点，认为年龄与围术期死亡率及长期生存率无关。一些国际权威指南也强调不应将高龄这单一因素视为手术禁忌。在老年患者中观察到增加的手术风险，可能与患者术前的合并症有关^[70, 71]^。对高龄患者应从术后肿瘤学效果和生理功能损失两方面进行权衡利弊，谨慎地做出判断。

目前普遍认为右全肺切除较左全肺切除具有更高的围术期死亡率^[13, 72-75]^。Fernandez等^[73]^研究结果表明，右全肺切除术的30 d、90 d死亡率为8%和16%，左全肺切除术为4%和9%，存在显著差异。Yang等^[75]^研究结果表明，右全肺切除是围术期90 d死亡的独立危险因素（相对危险度为2.23，*P* < 0.01）。这种差异可能由右全肺切除术后发生ARDS和BPF的概率较高引起。ARDS更常见于右全肺术后原因是肺功能损失较大，而且剩余左肺液体负荷更重，容易引起肺水肿。肺脏解剖学特征表明通常只有1条支气管动脉供应右主支气管，而2条或更多支气管动脉供应左主支气管，且右主支气管缺乏周围纵隔组织的保护，从而导致右全肺术后BPF的发生率较高。

BPF常常被用来评估是否为影响预后的危险因素。Stolz等^[76]^研究结果表明，BPF是影响围术期死亡及长期生存的独立危险因子。Janet-Vendroux等^[74]^研究结果表明，BPF及其他术后围术期并发症均是影响长期预后的独立危险因素。然而，Skrzypczak等^[14]^认为BPF不会影响患者的长期预后。目前大多数证据表明，BPF是影响患者的围术期生存的危险因素，尽可能防止BPF发生显得尤为重要。Zanotti等^[54]^表明BPF发生的危险因素包括肺良性疾病、营养不良、术前新辅助治疗，术前第一秒用力呼气容积（forced expiratory volume in the first second, FEV_1_）和肺一氧化碳弥散量（diffusion capacity for carbon monoxide, DLCO）降低、右全肺切除术、胸管拔除的时机。与缝线缝合相比，用吻合钉闭合支气管对支气管胸膜瘘有保护作用^[51]^。某些临床机构在术中常常进行支气管残端加固以降低支气管胸膜瘘的发生。有研究^[77]^通过*meta*分析发现，支气管残端加固处理可预防BPF的发生。Sfyridis等^[78]^进行的一项随机对照实验结果表明，采用肋间肌加固支气管残端可减少术后支气管胸膜瘘和脓胸的发生。有研究^[79]^表明支气管残端加固是支气管胸膜瘘和脓胸的危险因素。但此研究局限于单因素分析，而且作者仅对发生支气管胸膜瘘的高危患者术中行支气管残端加固，存在选择偏倚。

新辅助治疗在全肺切除中的应用存在一定争议。Bernard等^[47]^研究结果表明，术前放疗会显著增加围术期并发症发生率，而术前化疗会显著增加围术期死亡率。Shapiro等^[10]^研究结果表明，新辅助治疗后全肺切除患者的30 d死亡率是直接行全肺切除术的2倍。2009年，Albain等^[80]^进行的一项随机对照试验结果表明，全肺切除术前行新辅助放疗加化疗的患者的30 d死亡率高达26%。新辅助治疗后的全肺切除患者发生围术期30 d和90 d死亡大多数由呼吸系统并发症导致。其他原因还包括术后感染、心脏并发症等^[81]^。新辅助治疗因手术风险高和并发症发生率高而无法进行。

然而一些研究证明全肺切除术前新辅助治疗是安全的。Mansour等^[82]^研究结果表明，对于N2的患者术前新辅助化疗并没有增加围术期并发症发生率及死亡率，而且较直接手术具有更好的长期生存结果。Pricopi等^[11]^研究结果同样表明新辅助治疗并不是围术期90 d死亡的危险因素。而且术前新辅助治疗患者围术期90 d死亡率随着时间推移由20世纪80年代的21.9%降至现在的8.2%。这种改变并未在直接行全肺切除术的患者中体现。作者认为这种差异可能是由于选择偏倚造成的，只有非常适合的患者才在诱导治疗后行全肺切除术。有研究^[83]^对15例行术前新辅助治疗的全肺切除患者进行长期随访，虽然术后并发症发生率高达80.0%，但无围术期30 d死亡，且总体3年和5年生存率高达80.0%和57.1%。这些患者均是经过术前充分评估选择出来的，存在选择偏倚。而且他们对于术后并发症进行积极、仔细的管理，从而降低了围术期死亡率。现如今，NSCLC的治疗已从单一手术逐渐转变为多模式治疗。诱导疗法在治疗局部晚期NSCLC中已取得令人满意的效果。全肺切除术前行诱导治疗模式应用于某些适宜的患者是安全有效的。

随着腔镜技术发展，胸腔镜技术在肺叶切除中的应用逐渐普及。既往研究结果^[84-86]^显示，胸腔镜下肺叶切除术较传统开放手术具有术后并发症少、术后疼痛轻、住院时间短等更好的围术期结果和相同的生存预后。然而，胸腔镜下肺叶切除术的优势能否扩展到全肺切除术仍不明确。全肺切除术病例较少，导致手术医生胸腔镜经验相比肺叶切除术相对匮乏。所以胸腔镜下全肺切除术是否使患者受益仍不明确。目前大多数研究仅为病案报道或非病例对照研究。仅有少数研究比较胸腔镜与开放全肺切除术在治疗NSCLC中的疗效差异。Hennon等^[87]^第一次纳入多中心临床数据比较腔镜组与开放组围术期死亡率和5年生存率是否存在差异，其结果表明，均无统计学差异。2018年Yang等^[75]^第一次采用倾向评分匹配法比较腔镜组与开放组围术期结果及长期预后，研究结果表明：腔镜组手术时间更长，术中淋巴结清扫数目更多，术后拔管时间更短。在术中出血量、围术期并发症发生率和长期预后上两组无统计学差异。Nwogu等^[17]^的研究结果显示，腔镜组中位生存时间长于开放组，但腔镜组肿瘤小且分期较早，当作者基于病理分期行亚组分析时，这种差异消失。目前研究结果显示，胸腔镜下全肺切除术无论在围术期结果还是长期预后上至少不劣于开放全肺切除术，而且，在某些围术期指标上胸腔镜较开放手术更具优势。从长期预后来看，胸腔镜与开放术式具有相同的肿瘤学治疗效果。腔镜技术应用于全肺切除术安全可行的。未来进一步应开展多中心随机对照试验来进一步证明胸腔镜的优势及安全性。

除上述评估因素外，研究者往往还将性别、肺功能、吸烟史、肿瘤病理类型、肿瘤分期、术前合并症、是否扩大切除、术后辅助治疗等因素纳入风险评估中，这些因素也往往是影响预后的危险因素。

## 3 总结

随着现代医学水平的不断发展，全肺切除术的比例虽然明显下降，但作为一种独立术式，在特定情形下，仍具有独一无二的作用。全肺切除术作为独立的围术期并发症发生及死亡的危险因素，术前进一步明确相关危险因素，规避风险，可降低术后心肺并发症的发生，从而降低围术期并发症发生率及死亡率，为患者带来福音。
